# The clinical use of cryoprecipitate and fibrinogen concentrate: A scoping review

**DOI:** 10.1111/trf.70255

**Published:** 2026-05-19

**Authors:** Aaron S. Hess, Sandra K. White, Elizabeth P. Crowe, Jay S. Raval, Jennifer Andrews, Claudia S. Cohn, Mischa L. Covington, Melissa M. Cushing, Cyril Jacquot, Jenna Khan, Anil K. Panigrahi, Nabiha H. Saifee, Aaron A. R. Tobian, Mary M. McFarland, Simon J. Stanworth, Ryan A. Metcalf

**Affiliations:** ^1^ New Zealand Blood Service Christchurch New Zealand; ^2^ Department of Pathology University of Utah Salt Lake City Utah USA; ^3^ Department of Pathology Johns Hopkins University Baltimore Maryland USA; ^4^ Department of Pathology and Laboratory Medicine University of Vermont Burlington Vermont USA; ^5^ Department of Pathology, Microbiology, and Immunology Vanderbilt University Medical Center Nashville Tennessee USA; ^6^ Department of Pediatrics Vanderbilt University Medical Center Nashville Tennessee USA; ^7^ Department of Laboratory Medicine and Pathology University of Minnesota Minneapolis Minnesota USA; ^8^ Department of Pathology Mass General Brigham, Harvard University Boston Massachusetts USA; ^9^ Department of Pathology Weill Cornell Medicine, Cornell University New York New York USA; ^10^ Department of Pathology and Laboratory Medicine Children's National Hospital Washington District of Columbia USA; ^11^ Department of Pathology University of Virginia Charlottesville Virginia USA; ^12^ Department of Anesthesiology, Perioperative and Pain Medicine Stanford University Stanford California USA; ^13^ Department of Pathology Stanford University Stanford California USA; ^14^ Department of Laboratory Medicine and Pathology University of Washington Seattle Washington USA; ^15^ Department of Laboratory Medicine and Pathology Seattle Children's Hospital Seattle Washington USA; ^16^ University of Utah, Spencer S. Eccles Health Sciences Library Salt Lake City Utah USA; ^17^ John Radcliffe Hospital, Oxford University Hospitals NHS Foundation Trust Oxford UK; ^18^ NHS Blood and Transplant Oxford UK; ^19^ ARUP Laboratories Salt Lake City Utah USA

## Abstract

**Background:**

Acquired hypofibrinogenemia poses significant bleeding risks. Concentrated sources of fibrinogen, whether cryoprecipitate or fibrinogen concentrates, are widely used. This scoping review aimed to identify and map the available evidence on fibrinogen supplementation.

**Study Design and Methods:**

We used the JBI Manual and PRISMA‐ScR guidelines. We included patients of all ages treated with fibrinogen supplementation. The concept was treatment with cryoprecipitate or fibrinogen concentrate (prophylactically or therapeutically); and context was any clinical setting worldwide. Eligible studies included randomized trials and observational studies with comparator arms. Comprehensive searches of three databases were performed on February 21, 2025. Primary studies meeting inclusion criteria were selected. UpSet data visualizations displayed studies with intersecting sets of characteristics.

**Results:**

From 8181 references screened, 134 met inclusion criteria, of which 61 were randomized trials and 16 propensity‐matched observational cohort studies. The highest proportion of trials focused on cardiovascular surgery (28/61; 46%) then trauma (12/61; 20%) populations and mainly assessed use of fibrinogen therapeutically (34/61; 56%). More trials evaluated fibrinogen concentrate (53/61; 87%) compared to cryoprecipitate (8/61; 13%). Commonly reported outcomes included bleeding, transfusion needs, or mortality. Studies inconsistently reported dosing, timing, and definitions of hypofibrinogenemia. Studies evaluating certain key populations—including obstetrics and pediatrics—were limited. All but one propensity‐matched study was in either cardiovascular surgery or trauma.

**Discussion:**

There is a growing body of literature informing use of fibrinogen supplementation, particularly in cardiovascular surgery and trauma populations. Meta‐analyses appear feasible to inform evidence‐based guideline development; however, variability in dosing, timing, and definitions highlights the need for more harmonized research.

**Abbreviations**
DIC: disseminated intravascular coagulation
FC: fibrinogen concentrate
PICO: population, intervention, comparator, outcome
PRISMA‐ScR: Preferred Reporting Items for Systematic Reviews and Meta‐Analyses guidelines for scoping reviews
RCTs: randomized controlled trials
TBI: traumatic brain injuries
VET: viscoelastic testing

## 1 INTRODUCTION

Fibrinogen is a glycoprotein with a central role in hemostasis.^1^ Hypofibrinogenemia can be acquired due to disease, injury, consumption, or dilution, and is associated with poor patient bleeding outcomes.^2^ Common clinical situations associated with hypofibrinogenemia include major trauma, cardiac surgery, obstetric hemorrhage, liver disease, and disseminated intravascular coagulation (DIC).^3^ Prompt recognition and correction of hypofibrinogenemia are hypothesized to improve outcomes. However, the evidence regarding appropriate indications, dosing, and timing of fibrinogen supplementation has not been synthesized across all patient populations. One potential advantage of such an evidence synthesis would be to identify common relative effects, which are often constant in evidence‐based medicine.^4^

Clinicians usually treat acquired hypofibrinogenemia in high‐acuity environments such as operating rooms, emergency departments, and intensive care units. In these settings, bleeding may be multifactorial, and hypofibrinogenemia usually coexists with other coagulopathies. Laboratory measurement ascertaining fibrinogen levels—either by Clauss assay or viscoelastic testing—is not always feasible in real time, which may lead to empiric administration based on clinical judgment.^5^ Clinicians need a clearer understanding of when and how fibrinogen should be used, and whether particular strategies (e.g., timing, dose, formulation, etc.) offer important benefits to patients.

Despite the importance of fibrinogen in hemostasis, guidelines on the clinical use of fibrinogen supplementation are few and somewhat dated.^6^, ^7^ There are two primary interventions available: cryoprecipitate (a blood component) and fibrinogen concentrate (FC; a lyophilized factor pathogen‐inactivated preparation). There is also a commercial cryoprecipitate prepared from pathogen‐inactivated plasma (Intercept Fibrinogen Complex, Cerus Corporation, Concord, CA). Not all fibrinogen‐enriched products are universally available worldwide, and we lack clear consensus that they can be used interchangeably. Existing recommendations for use of fibrinogen supplementation vary across institutions and countries and are rarely based on high‐quality evidence.^8^, ^9^, ^10^ For example, in the United States, the choice between cryoprecipitate and FC is primarily decided at the hospital level and strongly influenced by relative cost and FDA approved indications. Furthermore, the literature on fibrinogen supplementation is varied, encompassing diverse patient populations, study designs, dosing strategies, and outcome measures.^11^ In developing this study, we identified nine prior systematic reviews and meta‐analyses of fibrinogen supplementation, none of which met our study aims.^5^, ^12^, ^13^, ^14^, ^15^, ^16^, ^17^, ^18^, ^19^ On this basis, we set out to determine whether there is enough research to make robust generalizations for a practical systematic review and meta‐analysis.

Our primary research aim was to conduct a rigorous scoping review on the use of cryoprecipitate or FC to treat or prevent bleeding, wherein we identify and map the sources of evidence. Our secondary aims were to assess the types of fibrinogen supplementation interventions tested, patient outcomes evaluated, and whether a systematic review and meta‐analysis could be conducted to support trustworthy clinical practice guidelines.

## 2 METHODS

The scoping review was conducted according to the JBI Manual for Evidence Synthesis and reported to the Preferred Reporting Items for Systematic Reviews and Meta‐Analyses guidelines for scoping reviews (PRISMA‐ScR, PRISMA‐S).^20^, ^21^, ^22^ A protocol was published on Open Science Foundation on February 19, 2025 (available at: https://doi.org/10.17605/OSF.IO/VWEAP).

### 2.1 Eligibility criteria

We framed our research question and criteria to JBI's mnemonic, “PCC”: Participants, Concept, and Context.^20^ Participants included patients of all ages or genders (a) treated with fibrinogen supplementation, or (b) at risk of bleeding, or (c) adverse events from fibrinogen supplementation, or (d) with acquired hypofibrinogenemia. Congenital hypofibrinogenemia was excluded. The concept included supplementation using either cryoprecipitate or FC and used prophylactically or for bleeding or non‐bleeding management. Also eligible in the original search were studies on acquired hypofibrinogenemia and their outcomes, regardless of whether fibrinogen was administered or using viscoelastic testing (VET). Due to the vastness of literature in the initial search, we chose to focus the scope of the review to full text articles that used FC or cryoprecipitate as an intervention. Fibrinogen levels were reviewed for study design, whether triggers of transfusion were evaluated, whether a fibrinogen intervention took place, and what impact fibrinogen treatment had on patient outcomes. The context was any clinical setting worldwide. Only primary research studies were eligible, including prospective and retrospective interventional studies where fibrinogen supplementation is given to at least a subset of study participants. We excluded all studies before 1971, the year cryoprecipitate was licensed for use in the United States.

### 2.2 Information sources

Three databases, Medline, Embase, and Cochrane, were searched on February 21, 2025. Further information on the search strategy is available in the Supporting Information.

No gray literature was included, nor were reference lists manually checked on included studies (forward citation searching) due to capacity constraints.

### 2.3 Study selection

Titles and abstracts were independently screened for relevance in Covidence by two of three investigators (SKW, ASH, EPC). Relevant studies identified received a full‐text review in the same fashion. Disagreements were resolved through discussion with another co‐author (RAM). No quality appraisal of included studies was performed, compliant with scoping review methodology.^20^ Two reviewers (SKW, RAM, or EPC) independently charted data using Covidence. Consensus was reached by discussion.

### 2.4 Data items

The PICO (population, intervention, comparator, outcome) framework was used to guide the data extraction form and incorporated the “PCC” framework as above. We considered different patient populations including trauma, obstetrics, orthopedics, liver disease, cardiovascular surgery, and pediatrics. Both cryoprecipitate and FC could be considered the intervention or comparison. Outcomes included mortality, bleeding, adverse events, laboratory values, further treatment or transfusions, and length of stay. We also recorded the type of care (therapeutic vs. prophylactic) and the clinical context of the intervention. Study characteristics including dates of study, country, and design were recorded. For each study, we indicated whether data were extractable for a future systematic review and/or meta‐analysis.

For randomized controlled trials (RCTs) and propensity‐matched studies, we extracted several additional variables. These included: whether timing of the intervention was reported, whether it was an early‐administration study (as defined by each study), fibrinogen dose given, and whether low fibrinogen was formally defined.

### 2.5 Statistical analysis

We tabulated and stratified results according to study design (overall, RCT, and propensity‐matched). To visualize patterns of overlap, we employed UpSet data visualizations, which provide a quantitative alternative to traditional Venn diagrams for representing intersections among categorical study characteristics.^23^, ^24^ UpSet plots were used to map co‐occurrence of key trial characteristics such as population, intervention type, study design, and outcomes.

Analyses were completed using R software. Differences between groups were assessed using chi‐square or the Fisher's exact test using the Bonferroni correction to adjust for multiple comparisons. *p*‐Values <.05 were considered statistically significant.

## 3 RESULTS

### 3.1 Overall literature search

**FIGURE 1:**
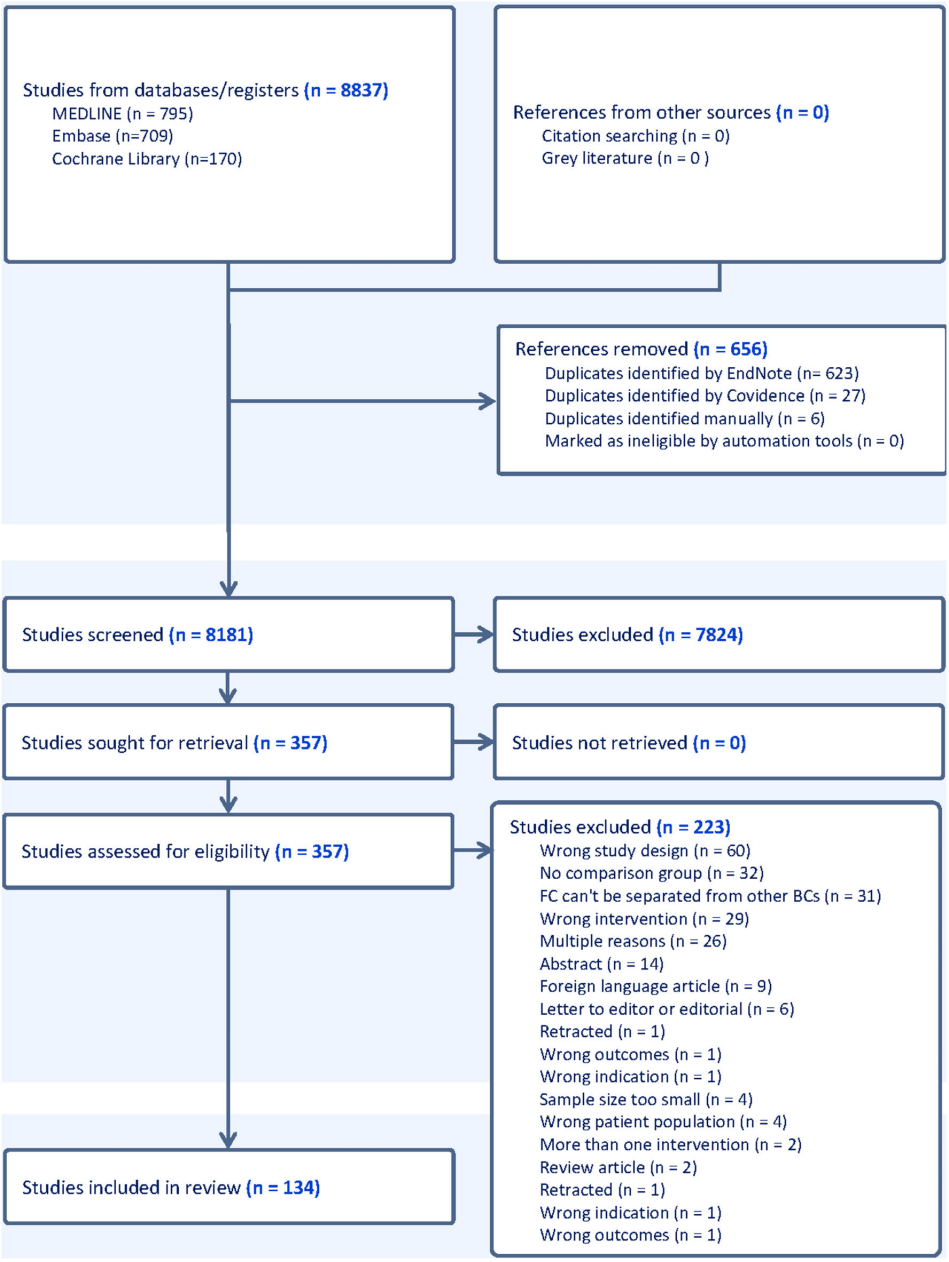
PRISMA diagram for study inclusion.

Database searches identified 8837 records, and after duplicates were removed 8181 were screened. We identified 357 publications for full‐text review. Of these, 134 met criteria for inclusion. The most common reasons for exclusion were ineligible study design (*n* = 60), lack of a comparison group (*n* = 32), or unable to separate FC from other blood components (*n* = 31) (Figure 1).

### 3.2 Included studies

Most studies were published in the United States (*n* = 18), United Kingdom (*n* = 15), Iran and Japan (*n* = 13 each) (Table 1). Almost half were published since 2020. Nearly all (92%) had data that could be extracted for a future meta‐analysis. Further details about individual study characteristics can be found in Table S1.

**TABLE 1:** Publication information for included studies.

Characteristic	Percentage (*n*)
Countries	
United States	13% (18/134)
United Kingdom	11% (15/134)
Iran	10% (13/134)
Japan	10% (13/134)
Multi‐country	7% (10/134)
Germany	6% (8/134)
Sweden	5% (7/134)
Other	37% (50/134)
Data sources	
Multiple publications using same data source	26% (35/134)
Secondary analysis of data	10% (13/134)
Subset of main study	4% (6/134)
Date of publication	
2020–present	46% (62/134)
2010–2019	46% (62/134)
2000–2009	4% (6/134)
1980–1999	3% (4/134)
Data extractable, one or more outcomes	92% (123/134)

**FIGURE 2:**
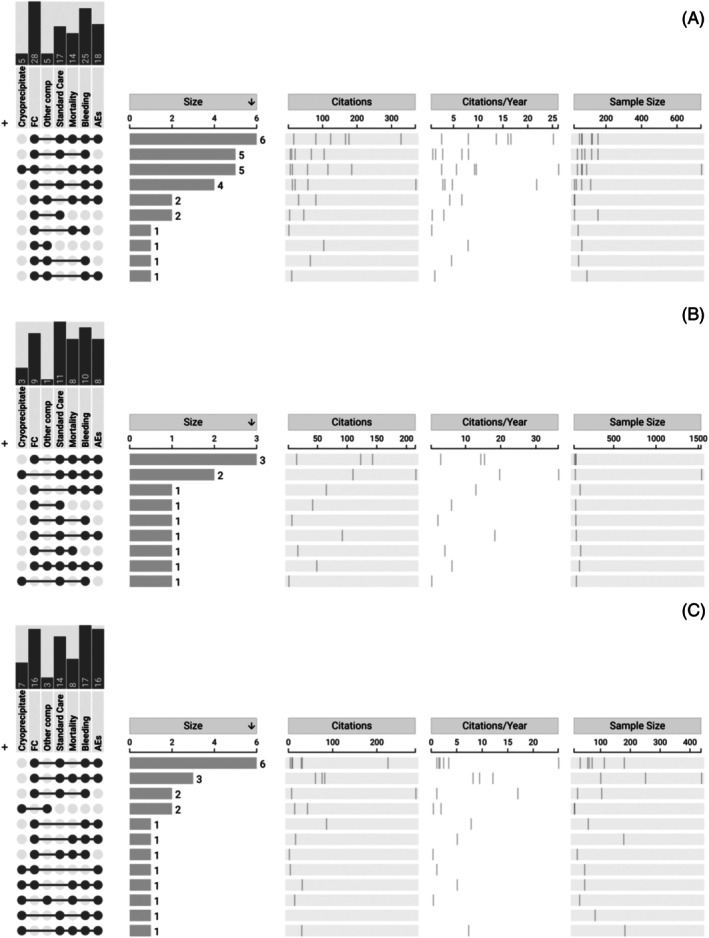
UpSet figures summarizing the interventions, comparators, outcomes, and other characteristics of randomized controlled trials included in this review. Studies are divided among (A) cardiac surgery, (B) trauma, and (C) all other populations, including obstetrics and pediatrics. The dot map on the left describes the most common patterns of interventions and outcomes. The first four columns indicate which fibrinogen intervention(s) were used: Cryoprecipitate, fibrinogen concentrate (“FC”), other (“other comp”), or standard care. The next three columns indicate which major outcomes were measured: mortality, bleeding, and adverse events (“AEs”). The bar chart surmounting this indicates the total number of times each characteristic appeared among the reviewed studies. To the right of the dot map, the horizontal bar chart (“Size”) indicates the number of studies that share that pattern, and the three barcode plots to the right indicate the number of total citations, citations per year, and sample sizes among the studies in the row that share the particular pattern of interventions and outcomes.

The most common populations studied were cardiovascular surgery and trauma (Table 2; Figure 2A,B). In general, cardiac studies were more likely to be RCTs, and trauma studies were more usually propensity‐matched cohorts. Most of the interventions focused on comparing cryoprecipitate or FC to standard care/placebo. Three‐quarters of all studies used FC as the intervention as opposed to cryoprecipitate, and among RCTs, FC was reported by almost all studies (87%). None of the identified studies included pathogen‐reduced cryoprecipitate. About two‐thirds of studies used FC and/or cryoprecipitate therapeutically (rather than prophylactically), and this was more common in propensity‐matched studies (81%, 13/16). Outcome measures varied significantly by design (Table 2).

**TABLE 2:** Characteristics of included studies^a^.

Characteristic	RCT percentage (*n*)	Propensity‐matched percentage (*n*)	Other percentage (*n*)	Overall percentage (*n*)
Study population				
Cardiovascular	48% (29/61)	31% (5/16)	28% (16/57)	37% (50/134)
Trauma^b^	20% (12/61)	63% (10/16)	39% (22/57)	33% (44/134)
Pediatrics	15% (9/61)	6% (1/16)	12% (7/57)	13% (17/134)
OB/GYN	7% (4/61)	0% (0/16)	12% (7/57)	8% (11/134)
Orthopedic	11% (7/61)	0% (0/16)	2% (1/57)	6% (8/134)
Transplant	3% (2/61)	0% (0/16)	9% (5/57)	5% (7/134)
Hematology/Oncology	7% (4/61)	0% (0/16)	4% (2/57)	4% (6/134)
General	0% (0/61)	6% (1/16)	7% (4/57)	4% (5/134)
Other	13% (8/61)	0% (0/16)	7% (4/57)	9% (12/134)
Intervention				
Fibrinogen concentrate^b^	87% (53/61)	63% (10/16)	65% (37/57)	75% (100/134)
Cryoprecipitate^b^	13% (8/61)	50% (8/16)	42% (24/57)	30% (40/134)
Comparator				
Standard care or placebo	69% (42/61)	88% (14/16)	65% (37/57)	69% (93/134)
Fibrinogen concentrate or cryoprecipitate	20% (12/61)	6% (1/16)	23% (13/57)	19% (26/134)
FFP	13% (8/61)	6% (1/16)	16% (9/57)	13% (18/134)
Other	2% (1/61)	0% (0/16)	12% (7/57)	6% (8/134)
Type of care				
Therapeutic	56% (34/61)	81% (13/16)	75% (43/57)	67% (90/134)
Prophylactic	31% (19/61)	13% (2/16)	12% (7/57)	21% (28/134)
Other	0% (0/61)	0% (0/16)	5% (3/57)	2% (3/134)
Not specified	13% (8/61)	19% (3/16)	12% (7/57)	13% (18/134)
Context				
Surgical^b^	69% (42/61)	19% (3/16)	35% (20/57)	49% (65/134)
Trauma^b^	18% (11/61)	63% (10/16)	40% (23/57)	33% (44/134)
Labor & Delivery	7% (4/61)	0% (0/16)	12% (7/57)	8% (11/134)
Critical care/ICU	5% (3/61)	13% (2/16)	5% (3/57)	6% (8/134)
Other	2% (1/61)	6% (1/16)	11% (6/57)	6% (8/134)
Outcomes				
Need for other blood products	79% (48/61)	44% (7/16)	63% (36/57)	68% (91/134)
Laboratory values^b^	82% (50/61)	13% (2/16)	63% (36/57)	66% (88/134)
Mortality^b^	49% (30/61)	100% (16/16)	67% (38/57)	63% (84/134)
Transfusion reactions/adverse events^b^	69% (42/61)	81% (13/16)	37% (21/57)	57% (76/134)
Volume of hemorrhage	57% (35/61)	25% (4/16)	32% (18/57)	43% (57/134)
Length of stay (ICU, hospital)	48% (29/61)	56% (9/16)	23% (13/57)	38% (51/134)
Surgical re‐exploration due to bleeding	34% (21/61)	19% (3/16)	14% (8/57)	24% (32/134)
Ventilator use	23% (14/61)	38% (6/16)	21% (12/57)	24% (32/134)
Achieved hemostasis	8% (5/61)	25% (4/16)	5% (3/57)	9% (12/134)
Bleeding scale	8% (5/61)	0% (0/16)	9% (5/57)	7% (10/134)
Other^c^	49% (30/61)	69% (11/16)	37% (21/57)	46% (62/134)

^a^ Percentages may add up to more than 100% since studies could report more than one category.

^b^ Statistically significant at *p* < .05 based on chi‐square or Fisher's exact test with the Bonferroni correction for multiple comparisons.

^c^ Other includes inotrope requirement, acute kidney injury or renal failure, sepsis, surgery duration, costs, and so forth.

Reported information on timing and dose of fibrinogen was inconsistent. Although nearly all studies reported dosing, only half of the studies reported information on timing of administration (Table 3). Approximately half of RCTs and slightly more than a third of propensity‐matched studies reported laboratory tests of fibrinogen at or before the timing of administration.

**TABLE 3:** Reporting by randomized controlled trials and propensity‐matched cohort studies.

Characteristic	RCT	Propensity‐matched
	Percentage (*n*)	Percentage (*n*)
Timing of fibrinogen/cryoprecipitate	52% (32/61)	50% (8/16)
Early administration	48% (29/61)	31% (5/16)
Dosage^a^	97% (59/61)	63% (10/16)
Low fibrinogen level defined	51% (31/61)	38% (6/16)

^a^ Statistically significant at *p* < .05 based on chi‐square or Fisher's exact test with the Bonferroni correction for multiple comparisons.

### 3.3 Description of populations

#### 3.3.1 Cardiovascular surgery

Among studies in cardiovascular surgery, the effect of fibrinogen on blood loss and subsequent transfusion requirements appeared inconsistent, but the designs of these studies varied greatly (Figure 2A). With notable exceptions, most trials recruited less than 100 participants, raising uncertainty about the ability of the trials to address clinically relevant questions with precision. However, for the most‐studied populations, the number and size of RCTs would still allow for meta‐analyses evaluating the impact of fibrinogen supplementation compared to standard care/placebo for key outcomes. In cardiovascular surgery, six and 15 trials using this study design reported mortality and bleeding outcomes, respectively. In trauma, seven trials reported on mortality, while nine reported bleeding outcomes.

Four trials compared preoperative prophylactic FC versus placebo.^25^, ^26^, ^27^, ^28^ Prophylactic doses of 1–2 g FC did not significantly reduce allogeneic transfusions,^25^, ^26^ nor did FC improve postoperative measures of fibrinogen function.^27^ Two of the trials reported small but statistically significant reductions in intraoperative blood loss; however, this finding should be interpreted cautiously, as intraoperative blood loss is inherently difficult to measure reliably.^25^, ^28^

Four additional trials studied intraoperative FC versus placebo.^29^, ^30^, ^31^, ^32^ Two trials compared intraoperative prophylaxis with FC to placebo with inconsistent results: 1 g FC reduced blood loss compared to placebo,^31^ while FC guided by VETs did not reduce transfusions.^30^, ^31^ Similarly, two trials compared empiric FC to placebo in response to intraoperative bleeding and observed no difference in blood loss^29^ or subsequent transfusion rates.^32^

Cryoprecipitate and FC were directly compared in two trials of patients with hypofibrinogenemia or bleeding after CPB. One trial randomized patients to 8–10 units of cryoprecipitate versus 1–2 g FC and observed no difference in fibrinogen levels, transfusion rates, or surgical re‐exploration.^33^ The second randomized 735 patients to FC (in 4 g doses) or cryoprecipitate (in 10‐unit doses) and found FC was noninferior with respect to transfusion requirements and thromboembolic events.^34^

Three unique surgical trials compared FC to different comparators. One trial randomized patients with bleeding after CPB to 4 g FC or one apheresis platelet unit.^35^ The FC arm received fewer subsequent platelet transfusions, but there were no differences in other blood products, laboratory tests, or clinical endpoints. A second trial randomized patients with massive bleeding to 4 units of plasma or 2 units of plasma plus 2 g FC and found that the plasma + FC arm had greater fibrinogen levels but worse plasma coagulation tests.^36^ A third trial randomized 116 patients at the end of CPB to either a VET‐guided dose of FC followed by prothrombin complex concentrates dosed 15 min later, or placebo at both time points, and found significantly fewer allogeneic transfusions in the intervention arm.^37^

Studies have also evaluated laboratory thresholds or the use of VET devices. Three trials tested laboratory‐guided FC during thoracoabdominal aneurysm repair. Laboratory‐guided doses of FC were associated with fewer subsequent transfusions than plasma^38^ or placebo.^39^ However, when the latter study was repeated in a multi‐center trial of 152 patients, the investigators observed unexpectedly higher rates of transfusion in the FC arm.^40^

In children, three trials in cardiovascular surgery compared laboratory‐guided therapy with FC to cryoprecipitate. Two of the studies found no difference in transfusion rates between patients randomized to FC or cryoprecipitate.^41^, ^42^ However, when one study was repeated by the investigators in a new, smaller cohort, there were significantly fewer allogeneic transfusions and better laboratory results in the FC arm.^43^

Two more trials in children compared FC versus placebo around the time of separation from CPB. One trial randomized infants to receive a weight‐based, laboratory guided dose of FC or placebo and observed significantly improved post‐dose laboratory measures in the FC arm.^44^ The second trial randomized participants to 70 mg/kg of prophylactic FC versus placebo at the end of CBP and observed an increased rate of rescue cryoprecipiate use in the placebo arm.^45^

Finally, one unique trial compared the effect of 70 mg/kg FC to 10 mL/kg of plasma on postoperative bleeding and clinical outcomes after congenital cardiac surgery and found smaller chest tube output volumes in the FC arm.^46^

#### 3.3.2 Trauma

Trauma studies generally observed no effect of early fibrinogen treatment on mortality, although the pooled sample size is dominated by a single large RCT of cryoprecipitate (Figure 2B).^47^ Three trials specifically included groups of patients with traumatic brain injuries (TBI) and found small, inconsistent benefits.^48^, ^49^, ^50^

Empiric treatment with fibrinogen in trauma was evaluated in four studies. In one large trial with 1604 participants, empiric treatment with three pools of cryoprecipitate did not affect 28‐day mortality or safety‐related outcomes compared to standard treatment,^47^ although a prespecified subgroup analysis suggested benefit with cryoprecipitate for patients with blunt injury. The pilot trial of the previous study had a similar design and similar results, although with a smaller population.^51^ Two small trials compared FC to placebo: one found no difference in 28‐day mortality or safety events,^52^ and the other found that prehospital FC improved fibrinogen measures, but lacked clinical outcome reporting.^53^

Three more trials tested fibrinogen supplementation following laboratory evidence of coagulopathy. One study directly compared FC to cryoprecipitate in 62 adult trauma patients with abnormal VET measures of fibrinogen function and found FC was associated with faster fibrinogen restoration but more deaths.^54^ However, this was a small pilot trial that was not powered for mortality. A second study in 90 patients found fewer deaths in an FC + RBC group compared to plasma + RBCs or RBCs alone.^55^ A third in 32 patients with hypofibrinogenemia by VET found FC was associated with shorter ICU stays than no treatment but no difference in transfusion rates.^56^

Treatment of TBI with fibrinogen was tested in three trials. In 54 patients with TBI and intracranial hemorrhage, four units of cryoprecipitate were associated with less hematoma expansion compared to no treatment,^50^ although the dosing and outcome definitions were unclear. A similar study (except with FC) in 137 TBI patients with fibrinogen levels <200 mg/dL observed better Glasgow Coma Scores at 24–72 h and less hematoma expansion in participants randomized to fibrinogen treatment.^49^ A third trial randomized TBI patients undergoing cranial surgery with fibrinogen <200 mg/dL to FC versus placebo and observed lower bleeding volumes but more frequent RBC transfusions in the FC arm.^48^

#### 3.3.3 Other populations

Additional key populations with RCTs included pediatrics, orthopedics, obstetrics, and hematology/oncology. Pediatric trials focused on either cardiovascular surgery or orthopedic populations and had sample sizes ranging from 30 to 111 subjects. All trials used FC as the primary intervention, although the comparators varied between studies (standard care, cryoprecipitate, or plasma). Bleeding outcomes were commonly reported, while mortality was less commonly reported, particularly in orthopedics.

Orthopedics trials in adults included three studies.^57^, ^58^, ^59^ One notable trial enrolled 178 patients undergoing hip arthroplasty and prophylactic FC was associated with smaller blood losses versus standard care.^59^ Obstetric populations included five randomized trials, with sample sizes ranging from 55 to 437.^60^, ^61^, ^62^, ^63^, ^64^ The two largest trials did not observe a benefit from the prophylactic use of FC to reduce the volume of post‐partum hemorrhage.^61^, ^64^ Among the smaller trials, one suggested possible lower RBC transfusion amounts with FC.^63^

Trials in hematology/oncology were all focused on surgical oncology populations and had sample sizes ranging from 21 to 70. The largest of these observed a trend toward improved bleeding‐related outcomes with FC versus placebo in patients undergoing radical cystectomy.^65^

## 4 DISCUSSION

There is a growing body of literature on fibrinogen supplementation for acquired hypofibrinogenemia. Identified studies evaluated a wide range of patient populations, including under‐researched groups such as children and pregnant women. Both cryoprecipitate and FC have been tested under trial conditions against each other (11/61; 18%), against plasma/other comparator (9/61; 15%), and against standard care/placebo (42/61; 69%). No clinical trial has evaluated pathogen‐reduced cryoprecipitate. Most studies included in this review reported clinical outcomes that are clearly important to patients, such as mortality or bleeding‐related outcomes (including subsequent blood product use, a possible surrogate for bleeding). We also observed that the pace of research is increasing: over 90% of identified studies were published in the last 15 years, with half published in the last 5 years.

### 4.1 Summary of evidence

We believe there is sufficient literature for a systematic review with focused meta‐analyses to support new guidelines. Important PICO questions on the use of therapeutic fibrinogen in cardiovascular surgery and trauma could be addressed, although the certainty of evidence is not yet clear. Specific clinical questions in pediatric cardiovascular surgery may also be evaluable based on the existing literature.

Despite the widespread availability of cryoprecipitate in countries such as the US and UK, FC seems overrepresented in the available data. This may be related to a higher proportion of trials being industry‐funded, although such an assessment was not performed in this review. Only 30% of the overall studies used cryoprecipitate, and only 13% of RCTs used cryoprecipitate as a study intervention. Both cryoprecipitate and FC were developed in the 1960s, but FC was not commercially available in most countries until the 21st century.^66^ The trials we reviewed which compared FC and cryoprecipitate suggest that they are roughly equivalent in most clinical applications, but that conclusion across populations must be considered preliminary.

Although available research on fibrinogen supplementation touches on a wide range of patients, most of it is concentrated in two areas and one intervention. Most of the identified studies were RCTs in either cardiovascular surgery or trauma, in which FC was used empirically to treat bleeding. There were few identified studies in general medical or hematology/oncology populations, and relatively few in pediatrics, liver transplant, or obstetrics. This fact is in stark contrast to real world North American studies that have shown that 61% of labor and delivery units surveyed had FC readily available for use.^67^ Differences in bleeding phenotypes of these populations; for example, obstetrics versus pediatric cancer, could mean it is difficult to draw broad conclusions from these limited and specialized data.^2^ Most of the studies did not define or rigorously evaluate laboratory thresholds for administration of fibrinogen therapies, and inclusion of patients without hypofibrinogenemia may have skewed results.

It is difficult to study treatments for acquired hypofibrinogenemia, for example in the context of bleeding. The disease presents unpredictably, making it time‐consuming and expensive to run trials, and usually occurs in environments like the emergency department and the operating room where enrollment, measurement, blinding, and control are difficult. Conventional cryoprecipitate is hard to use in trial situations because it takes time to thaw and is only usable for up to 6 h post‐thaw before it must be discarded unless pathogen‐reduced. As fibrinogen is often administered with other blood components, its relative effect on outcomes may be obscured. Nevertheless, the number of trials in this space has increased significantly in recent years.

About half of the included RCTs investigated “early” fibrinogen administration, typically defined by clinical bleeding rather than laboratory confirmation of low fibrinogen levels. The definition of “early” differed widely among studies, and in many cases, treatment was initiated more than 1–3 h after bleeding onset. Half of the RCTs also did not define “low” fibrinogen in the trial population. This approach to trial design is common and may be pragmatic, intended to reflect how fibrinogen is used at the bedside. But the causal link between fibrinogen and important outcomes—mortality, blood loss, blood product use, thrombotic events—may be co‐dependent on other interventions such as platelet transfusions, coagulation factors, and mechanical control of bleeding. Even if a pragmatic trial proves the utility of early fibrinogen in a specific situation, it may be challenging to adapt those results to other populations when the definitions of hypofibrinogenemia are not rigorously defined and the indications for administration are not based on readily reproducible measures.

### 4.2 Limitations

This review has some limitations. Despite searches of three major research databases, relevant published studies may have been missed. Our Medline strategy was the most sensitive and comprehensive search across all of our research questions, whereas Embase and Cochrane Library searches were developed for precision to concepts from the primary research question. We did not search gray literature, sources outside commercial databases, nor did we conduct citation analysis for our included studies. Although we used multiple reviewers to screen articles and the full text of candidates, it is possible that potentially informative studies were mistakenly excluded from our review. Finally, we were only able to screen manuscripts in English and may have missed relevant research in other languages.

### 4.3 Conclusion

In conclusion, we found that there is an increasing body of evidence on the use of fibrinogen therapies in acquired hypofibrinogenemia, particularly in populations of cardiovascular surgery and trauma. Given the variety of study designs, intervention strategies, and reporting of outcomes, future broad meta‐analyses assessing common relative effects across patient populations may present challenges. As the use of fibrinogen in clinical practice increases, and as FC and newer products such as pathogen‐reduced cryoprecipitate become more widely available, clinicians and policymakers need to be able to understand the available evidence and may benefit from evidence‐based guidelines. Charting the current landscape of evidence on fibrinogen supplementation may be helpful to guide future research to support evidence‐based management of acquired hypofibrinogenemia with the goal of improving important patient outcomes.

## CONFLICT OF INTEREST STATEMENT

RAM reports scientific advising for Cerus Corporation and Werfen in the past. He reports having received grants from Werfen, Haemonetics, Haemosonics, and Octapharma, which are no longer active. EPC reports personal fees from Plas‐Free Ltd. and Terumo BCT outside of the submitted work. JA reports receiving research grants from Cerus Corporation and scientific advising for Octapharma. MC reports receiving a research grant from Cerus Corporation and scientific advising for Cerus, Octapharma, Grifols, CSL Behring, Werfen, and Haemonetics. AKP reports scientific advising for Octapharma. AART reports personal fees and non‐financial support from Grifols, Ortho Clinical Diagnostics, and Teleflex outside of the submitted work. He is also the PI of a US government funded clinical trial of pathogen reduction.

## Supporting information


**Data S1.** Supporting Information 1.

**Data S2.** Supporting Information 2.

## Data Availability

The data that support the findings of this study are available from the corresponding author upon reasonable request.
