# Supplementary material for: The clinical use of cryoprecipitate and fibrinogen concentrate: A scoping review
Source: Transfusion. 2026 May 19;66(7):1424–37. doi: 10.1111/trf.70255 (PMC13350501; doi:10.1111/trf.70255)
Supplement: Supplementary file 2 — Data S2. Supporting Information 2. [file TRF-66-1424-s002.docx]

**Supplemental Materials**

## Search

An information specialist (MMM) developed the search strategies using a combination of database subject terms and keywords harvested from sentinel articles and team feedback for each of our concepts: fibrinogen supplementation, hypofibrinogenemia, and viscoelastic testing (VET) studies where a fibrinogen surrogate marker was evaluated or used to guide fibrinogen supplementation therapy decisions. No date limits nor methodological filters were used. An animal filter was utilized to limit to humans in Medline and Embase. A library colleague from the Evidence Review Team peered review the strategy according to PRESS guidelines.^63^ See Supplement A for full search details. EndNote, version 21 (Clarivate) was used for citation management and duplicate article detection and removal. Covidence (Veritas Health Innovation), the online systematic reviewing platform, provided a second pass for identifying duplicates.

**Supplemental Table 1**. Individual characteristics of randomized controlled trial and propensity-matched studies

| **Study** | **Country** | **Study period** | **Study design** | **Population** | **Type of intervention** |
| --- | --- | --- | --- | --- | --- |
| Ahmed 2012 | Ireland | 2009-2011 | Prospective cohort | Obstetrics | Therapeutic |
| Akbari 2018 | Iran | 2015-2016 | RCT | Trauma | Therapeutic |
| Almskog 2020 | Sweden | 2013-2015 | Propensity-matched | Trauma | Therapeutic |
| Araki 2015 | Japan | 2010-2012 | Retrospective cohort | Cardiovascular | Therapeutic |
| Ayaganov 2023 | Kazakhstan | 2021-2022 | RCT | Cardiovascular | Therapeutic |
| Balvers 2017 | Multiple | 2008-2015 | Prospective cohort | Trauma | Therapeutic |
| Barquero Lopez 2022 | Spain | 2008-2019 | Retrospective cohort | Trauma | Empiric |
| Baryshnikova 2024 | Italy | 2023-2024 | RCT | Cardiovascular | Therapeutic |
| Bilecen 2013 | Netherlands | 2007-2010 | Prospective cohort | Cardiovascular | Therapeutic |
| Bilecen 2017 | Netherlands | 2011-2015 | RCT | Cardiovascular | Therapeutic |
| Brodin 1985 | Sweden | Unknown | RCT | Infectious disease | Not specified |
| Budnick 2021 | United States | 2011-2018 | Retrospective cohort | Liver ICU | Other |
| Buechner 2021 | Multiple | Unknown | Unknown | Hematology/Oncology | Prophylactic |
| Caballero 2023 | Spain | 2019-2021 | RCT | Transplant | Therapeutic |
| Callum 2019 | Canada | 2017-2018 | RCT | Cardiovascular | Therapeutic |
| Chen 2020 | China | 2017-2019 | RCT | Pediatrics, Orthopedics | Not specified |
| Clayton 1995 | United States | 1990-1991 | Unknown | Transplant | Therapeutic & Prophylactic |
| Collins 2017 | United Kingdom | 2013-2015 | RCT | Obstetrics | Therapeutic |
| Curry 2015 | United Kingdom | 2012-2013 | RCT | Trauma | Therapeutic |
| Curry 2018 | United Kingdom | 2016 | RCT | Trauma | Therapeutic |
| Davenport 2023 | Multiple | 2017-2021 | RCT | Trauma | Therapeutic |
| Deleu 2022 | France | 2012-2013 | Prospective cohort | Obstetrics | Therapeutic |
| Ditillo 2020 | United States | 2015-2016 | Retrospective cohort | Trauma | Therapeutic |
| Dorken-Gallastegi 2023 | United States | 2013-2019 | Retrospective cohort | Trauma | Therapeutic |
| Downey 2020 | United States | 2016-2018 | RCT | Pediatrics, Cardiovascular | Therapeutic |
| Downey 2024 | United States | 2019-2021 | RCT | Pediatrics, Cardiovascular | Not specified |
| Ducloy-Bouthors 2021 | France | 2014-2018 | RCT | Obstetrics | Therapeutic |
| Edwards 2018 | Denmark | 2011-2013 | RCT | Obstetrics | Therapeutic |
| Endo 2022 | Multiple | 2015-2019 | Propensity-matched | Trauma | Therapeutic |
| Fassl 2015 | Switzerland | 2008-2009 | Propensity-matched | Cardiovascular | Therapeutic |
| Fathi 2021 | Iran | 2019-2020 | RCT | Hematology/Oncology | Prophylactic |
| Fenger-Eriksen 2009 | Denmark | 2007-2008 | RCT | Hematology/Oncology | Therapeutic |
| Fleming 2022 | United States | 2017-2020 | Propensity-matched | Trauma | Therapeutic |
| Fletcher 2024 | Australia | 2005-2021 | Retrospective cohort | Cardiovascular | Therapeutic |
| French 2003 | Australia | Unknown | RCT | Liver disease | Prophylactic |
| Fujiwara 2023 | Japan | 2016-2021 | Retrospective cohort | Trauma | Not specified |
| Gaitanidis 2022 | United States | 2017 | Propensity-matched | Trauma | Therapeutic |
| Galas 2014 | Brazil | Unknown | RCT | Pediatrics, Cardiovascular | Therapeutic |
| Gerard 2023 | United States | 2017-2021 | Retrospective cohort | Pediatrics, Trauma | Therapeutic |
| Giordano 2019 | Italy | 2011-2013 | Retrospective cohort | Pediatrics,  Hematology/Oncology | Prophylactic |
| Gollop 2012 | United Kingdom | 2008-2010 | Prospective cohort | General | Therapeutic & Prophylactic |
| Gonzalez-Guerrero 2017 | Spain | 2012-2014 | Retrospective cohort | Trauma | Therapeutic |
| Green 2022 | United Kingdom | 2019-2020 | RCT | Obstetrics | Therapeutic |
| Guan 2023 | China | 2020-2021 | Prospective cohort | Cardiovascular | Therapeutic |
| Haas 2015 | Switzerland | 2012-2014 | RCT | Pediatrics, Orthopedics | Prophylactic |
| Hamada 2020 | France | 2012-2018 | Prospective cohort | Trauma | Therapeutic |
| Hartmann 2019 | Germany | 2007-2011 | Retrospective cohort | Transplant | Therapeutic |
| Heo 2024 | South Korea | 2015-2022 | Propensity-matched | Trauma | Therapeutic |
| Hesselvik 1985 | Sweden | Unknown | RCT | Septic ICU | Not specified |
| Hinton 2023a | Australia | 2005-2018 | Propensity-matched | Cardiovascular | Not specified |
| Hinton 2023b | Australia | 2001-2019 | Propensity-matched | Cardiovascular | Therapeutic & Prophylactic |
| Holcomb 2013 | United States | 2009-2010 | Prospective cohort | Trauma | Therapeutic |
| Horst 2023 | United States | 2014-2018 | Observational, Prospective cohort | General Pediatrics | Therapeutic |
| Inokuchi 2017 | Japan | 2011-2015 | Retrospective cohort | Orthopedics | Therapeutic |
| Ismail 2022 | Egypt | 2020-2022 | RCT | Orthopedics | Prophylactic |
| Itagaki 2020 | Japan | 2010-2018 | Propensity-matched | Trauma | Not specified |
| Jahangirifard 2018 | Iran | 2012-2014 | Retrospective cohort | Transplant | Prophylactic |
| Javaherforoosh Zadeh 2019 | Iran | 2018-2019 | RCT | Cardiovascular | Prophylactic |
| Jeppsson 2016 | Sweden | 2009-2012 | RCT | Cardiovascular | Prophylactic |
| Kamidani 2021 | Japan | 2013-2020 | Retrospective cohort | Obstetrics | Therapeutic |
| Karlsson 2009 | Sweden | 2006 | RCT | Cardiovascular | Prophylactic |
| Karlsson 2011 | Sweden | 2006 | RCT | Cardiovascular | Prophylactic |
| Khalaf-Adeli 2019 | Iran | 2017 | RCT | Cardiovascular | Prophylactic |
| Kikura 2023 | Japan | 2010-2019 | Retrospective cohort | Cardiovascular | Not specified |
| Kim 2023 | South Korea | 2021-2022 | Retrospective cohort | Transplant | Not specified |
| Kwapisz 2020 | Canada | 2013-2019 | RCT | Cardiovascular | Prophylactic |
| Lance 2012 | Netherlands | Unknown | RCT | Cardiovascular, Orthopedics, Abdominal surgery | Therapeutic |
| Leal-Noval 2014 | Spain | 2011-2013 | Propensity-matched | General w/massive transfusion protocol activation | Therapeutic |
| Liu 2013 | China | 2003-2010 | Retrospective cohort | Transplant | Not specified |
| Lubkin 2024 | United States | 2017-2021 | Retrospective cohort | Trauma | Therapeutic |
| Lucena 2021 | Brazil | 2015-2017 | RCT | Trauma | Therapeutic |
| Machotta 2021 | Netherlands | 2012-2016 | RCT | Pediatrics, Orthopedics | Prophylactic |
| Maeda 2019 | Japan | 2010-2012 | Propensity-matched | Cardiovascular | Therapeutic & Prophylactic |
| Mallaiah 2015 | United Kingdom | 2011-2013 | Prospective cohort | Obstetrics | Therapeutic |
| Massoumi 2018 | Iran | 2014-2015 | RCT | Pediatrics, Cardiovascular | Therapeutic |
| Matsunaga 2017 | Japan | 2004-2016 | Retrospective cohort | Obstetrics | Therapeutic |
| Miller 1997 | United States | Unknown | Observational | Pediatrics, Cardiovascular | Therapeutic |
| Morrison 2013 | United States, United Kingdom | 2006-2011 | Retrospective cohort | Trauma | Therapeutic |
| Morrison 2019 | United Kingdom | 2010-2013 | RCT | Cardiovascular | Prophylactic |
| Najafi 2014 | Iran | 2011-2013 | RCT | Orthopedics | Prophylactic |
| Nakajima 2023 | Japan | 2016-2022 | Retrospective cohort | Obstetrics | Therapeutic |
| Nascimento 2016 | Canada | 2014-2015 | RCT | Trauma | Therapeutic |
| Niakan 2023 | Iran | 2018-2019 | RCT | Traumatic brain injury | Not specified |
| Obaid 2022 | United States | 2016-2017 | Propensity-matched | Trauma | Not specified |
| Olaussen 2016 | Australia | 2008-2011 | Retrospective cohort | Trauma | Therapeutic |
| Peng 2019 | Canada | 2014-2015 | RCT | Trauma | Therapeutic |
| Ponschab 2015 | Austria | 2008-2015 | Retrospective cohort | Trauma | Therapeutic |
| Rahe-Meyer 2009a | Germany | 2006 | Prospective cohort, Retrospective cohort | Cardiovascular | Therapeutic |
| Rahe-Meyer 2009b | Germany | 2006 | Prospective cohort, Retrospective cohort | Cardiovascular | Therapeutic |
| Rahe-Meyer 2013a | Germany | 2008-2010 | RCT | Cardiovascular | Therapeutic |
| Rahe-Meyer 2013b | Germany | 2008-2010 | RCT | Cardiovascular | Therapeutic |
| Rahe-Meyer 2016 | Multiple | 2012-2014 | RCT | Cardiovascular | Therapeutic |
| Rahe-Meyer 2019 | Multiple | 2012-2014 | RCT | Cardiovascular | Therapeutic |
| Rahe-Meyer 2021 | Multiple | 2012-2014 | RCT | Cardiovascular | Therapeutic |
| Ranucci 2015 | Italy | 2011-2014 | RCT | Cardiovascular | Not specified |
| Ranucci 2016 | Italy | 2011-2014 | RCT | Cardiovascular | Not specified |
| Rizza 2024 | Italy | 2020-2022 | Retrospective cohort | Pediatrics, Cardiovascular | Therapeutic |
| Rourke 2012 | United Kingdom | 2008-2010 | Prospective cohort | Trauma | Therapeutic |
| Roy 2020 | United Kingdom | 2017-2018 | RCT | Hematology/Oncology | Therapeutic |
| Roy 2023 | United Kingdom | 2017-2018 | RCT | Hematology/Oncology | Therapeutic |
| Sabate 2016 | Spain | 2012-2014 | RCT | Transplant | Prophylactic |
| Sabouri 2022 | Iran | 2019-2021 | RCT | Traumatic brain injury | Not specified |
| Sadeghi 2014 | Iran | Unknown | RCT | Cardiovascular | Prophylactic |
| Saeidiborojeni 2022 | Iran | Unknown | RCT | Traumatic brain injury | Therapeutic |
| Schlimp 2016 | Austria | 2005-2015 | Retrospective cohort | Trauma | Therapeutic |
| Schochl 2014 | Austria | 2010-2011 | Prospective cohort | Trauma | Therapeutic |
| Shakeri 2023 | Iran | 2022 | RCT | Orthopedics | Prophylactic |
| Shams-Hakimi 2019 | Sweden | Unknown | Observational | Cardiovascular | Therapeutic |
| Shang 2024 | China | 2013-2017 | Propensity-matched | Trauma | Therapeutic |
| Shaz 2010 | United States | 2005-2009 | Prospective cohort, Retrospective cohort | Trauma | Therapeutic |
| Shibahashi 2019 | Japan | 2011-2016 | Retrospective cohort | Traumatic brain injury | Empiric |
| Siemens 2020 | United Kingdom | 2014-2016 | RCT | Pediatrics, Cardiovascular | Prophylactic |
| Siemens 2023 | United Kingdom | 2014-2016 | Observational, Prospective cohort | Pediatrics, Cardiovascular | Not specified |
| Siemens 2025 | United Kingdom | 2014-2016 | RCT, Prospective cohort | Pediatrics, Cardiovascular | Not specified |
| Soleimani 2017 | Iran | 2014-2015 | RCT | Urology | Prophylactic |
| Solomon 2012 | Germany | 2006 | Prospective cohort | Cardiovascular | Therapeutic |
| Solomon 2013 | Germany | 2008-2010 | RCT | Cardiovascular | Therapeutic |
| Stinger 2008 | Iraq | 2004-2005 | Retrospective cohort | Trauma | Therapeutic |
| Sugiyama 2020 | Japan | 2013-2018 | Retrospective cohort | Traumatic brain injury | Therapeutic |
| Tama 2021 | Multiple | 2014-2017 | Propensity-matched | Pediatrics, Trauma | Therapeutic |
| Tanaka 2014 | United States | Unknown | RCT | Cardiovascular | Therapeutic |
| Theodoulou 2012 | United Kingdom | Unknown | Retrospective cohort | General | Therapeutic |
| Tirotta 2022 | United States | 2017-2018 | RCT | Pediatrics, Cardiovascular | Prophylactic |
| Vigstedt 2022 | Denmark | 2015-2016 | Retrospective cohort | General | Therapeutic |
| Vlot 2022 | Netherlands | 2014-2018 | RCT | Cardiovascular | Therapeutic |
| Wafaisade 2013 | Germany | 2005-2010 | Propensity-matched | Trauma | Therapeutic |
| Walden 2020 | Sweden | 2009-2014 | Propensity-matched | Cardiovascular | Therapeutic |
| Winearls 2021 | Australia | 2016-2017 | RCT | Trauma | Therapeutic |
| Yamamoto 2014 | Japan | Unknown | Retrospective cohort | Cardiovascular | Prophylactic |
| Yamamoto 2016 | Japan | 2011-2015 | Retrospective cohort | Trauma | Therapeutic & Prophylactic |
| Yan 2024 | China | 2021 | Retrospective cohort | Obstetrics | Therapeutic |
| Yang 2013 | United Kingdom | 2004-2010 | Retrospective cohort | Cardiovascular | Therapeutic |
| Ziegler 2021 | Multiple | 2011-2015 | RCT | Trauma | Therapeutic |
| Zrimsek 2024 | Slovenia | 2018-2023 | Interventional | Therapeutic plasma exchange | Not specified |

ICU: Intensive care unit, RCT: Randomized controlled trial
